# The clinical significance of the *FUS-CREB3L2 *translocation in low-grade fibromyxoid sarcoma

**DOI:** 10.1186/1749-799X-6-15

**Published:** 2011-03-15

**Authors:** Barry Rose, George S Tamvakopoulos, Kamaljit Dulay, Robin Pollock, John Skinner, Timothy Briggs, Steven Cannon

**Affiliations:** 1 The Royal National Orthopaedic Hospital NHS Trust, The London Bone and Soft-Tissue Tumour Unit, London, UK; 2 The Royal National Orthopaedic Hospital NHS Trust, Department of Histopathology, London, UK

## Abstract

**Background:**

Low-grade fibromyxoid sarcoma (LGFMS) is a rare soft-tissue neoplasm with a deceptively benign histological appearance. Local recurrences and metastases can manifest many years following excision. The *FUS-CREB3L2 *gene translocation, which occurs commonly in LGFMS, may be detected by reverse-transcriptase polymerase chain reaction (RT-PCR) and fluorescence in situ hybridisation (FISH). We assessed the relationship between clinical outcome and translocation test result by both methods.

**Methods:**

We report genetic analysis of 23 LGFMS cases and clinical outcomes of 18 patients with mean age of 40.6 years. During follow-up (mean 24.8 months), there were no cases of local recurrence or metastasis. One case was referred with a third recurrence of a para-spinal tumour previously incorrectly diagnosed as a neurofibroma.

**Results:**

Results showed 50% of cases tested positive for the *FUS-CREB3L2 *translocation by RT-PCR and 81.8% by FISH, suggesting FISH is more sensitive than RT-PCR for confirming LGFMS diagnosis. Patients testing positive by both methods tended to be younger and had larger tumours. Despite this, there was no difference in clinical outcome seen during short and medium-term follow-up.

**Conclusions:**

RT-PCR and FISH for the *FUS-CREB3L2 *fusion transcript are useful tools for confirming LGFMS diagnosis, but have no role in predicting medium-term clinical outcome. Due to the propensity for late recurrence or metastasis, wide excision is essential, and longer-term follow-up is required. This may identify a difference in long-term clinical outcome between translocation-positive and negative patients.

## Background

Low-grade fibromyxoid sarcoma (LGFMS) is a rare low-grade neoplasm first described in 1987 [1]. Its true incidence is unknown. LGFMS most commonly affects young to middle-aged adults, and has a male: female ratio of approximately 3:1 [2-4]. Typically LGFMS occurs in the trunk and proximal extremities, and lies deep to fascia, although it may occur superficially [1-3,5]. It usually presents as a painless mass. There have been case reports of the tumour arising intra-cranially [6,7], within the thoracic cavity [8] and abdominal cavity [9].

LGFMS is an indolent tumour with a deceptively benign histological appearance [1,10]. The diagnosis of LGFMS by histopathology alone may not be easily reached because of the bland appearance of the sections, which can resemble other benign or low-grade fibromyxoid lesions [11]. Molecular testing can be used to aid or confirm the diagnosis. Local recurrences are not uncommon (≤10%), and the tumour metastasises in 5-10% of cases [5,12]. Despite this, there is good long-term survival [1]. Treatment is by surgical excision. LGFMS has not been found to be chemo- or radio-sensitive.

The characteristic chromosomal translocation t(7;16)(q33;p11) results in the balanced *FUS-CREB3L2 *fusion gene, which has been shown to be present in most cases of LGFMS [10,11,13-16]. The translocation t(11,16)(p11;p11) results in the balanced *FUS-CREB3L1 *fusion gene, which is also found in cases of LGFMS, though less frequently [13,14]. Cytogenetic and molecular genetic approaches can, therefore, be used as a tool for arriving at a diagnosis of LGFMS [15,17].

The tumour-specific *FUS-CREB3L2 *fusion gene can be detected using a reverse-transcription polymerase chain reaction (RT-PCR) using formalin-fixed paraffin-embedded tissue [11,18] (the sensitivity using DNA-based PCR is reported to be lower [18]), and by fluorescence in situ hybridisation (FISH).

The aim of this paper is to review our series of patients with LGFMS to assess the relationship between *FUS-CREB3L2 *test results using RT-PCR and FISH, and to assess any correlation these may have with the clinical outcome.

## Materials & methods

A retrospective review of our histopathological database was carried out. All patients with a diagnosis of LGFMS, as classified by the World Health Organisation, were included in the study [19]. Our study covered the period 2004-2008.

All patients underwent pre-operative Magnetic Resonance Imaging (MRI), and the diagnosis was reached on a needle core biopsy which was followed by wide local excision, or on excision biopsy. Staging was achieved with a Computed Tomography (CT) scan of the chest and a technetium-99 bone scan.

Outcome measures included time to local recurrence, presence/absence of metastases and survival data. All resection specimens were subjected to conventional analysis including tumour margins. They were all subjected to cytogenetic analysis by RT-PCR and FISH. *FUS-CREB3L2 *RT-PCR was performed by RNA extraction (Ambion) from paraffin-embedded tumour blocks. This was followed by RNA quantification, reverse transcription and addition of specific primer (Invitrogen) to obtain a PCR product. The PCR product was then visualised on an agrose gel. FISH analysis was performed using bacterial artificial chromosome (BAC) clones that were selected according to their location (chromosomes 7 and 16) with regard to the genes involved in the *FUS-CREB3L2 *translocation. The clones were prepared, processed, labelled and analysed for fluorescent signals according to standard procedures. The presence or absence of the *FUS-CREB3L2 *translocation by both methods was noted.

Histopathological analysis and clinical outcomes for all identified cases of LGFMS were compared to results of *FUS-CREB3L2 *translocation PCR and FISH testing and subjected to statistical analysis using the Student's T test.

## Results

Twenty-three tumours were identified in 23 patients. Five cases consisted of slides referred from other units for our specialist opinion. There were 10 male and 13 female patients. The mean age was 40.6 years (range 14-70 years). Ten tumours (43.5%) were located in the lower limb, 5 (21.7%) in the upper limb, and 7 (30.4%) were related to the trunk, and the location of 1 tumour (4.3%) was not specified. The history of symptom duration was available in 14 patients, with a mean of 33.3 months (range 3-300 months).

All 5 referred cases were resection specimens. Of the 18 cases from our institution, 14 had a pre-operative core needle biopsy (77.8%), and 4 went straight on to excision biopsy (22.2%). All 18 of these patients were treated definitively with surgical excision. In all cases that were biopsied, LGFMS was correctly diagnosed by histopathological examination prior to resection.

Excision was wide in 6 resection cases (33.3%) and marginal in 12 cases (66.7%). Marginally excised cases all had a 1 mm margin of normal tissue. There were no cases of intra-lesional excision. The mean maximum diameter of the resected tumours was 84.3 mm (range 20-150 mm).

Three patients were lost to follow-up. Mean follow-up of the remaining 15 cases was 24.8 months following surgical excision (range 6 to 53 months). One case was referred as recurrence of a para-spinal neurofibroma that had previously been resected 9 years and subsequently 5 years prior to referral. Biopsy histology from the lesion suggested a malignant peripheral nerve sheath tumour, but histology from our resection revealed a LGFMS. She underwent subsequent radiotherapy, and has had no further recurrence. No other patients received chemotherapy or radiotherapy at any point. Apart from the above patient there were no cases of local recurrence. There were no cases of metastasis.

**Figure 1:**
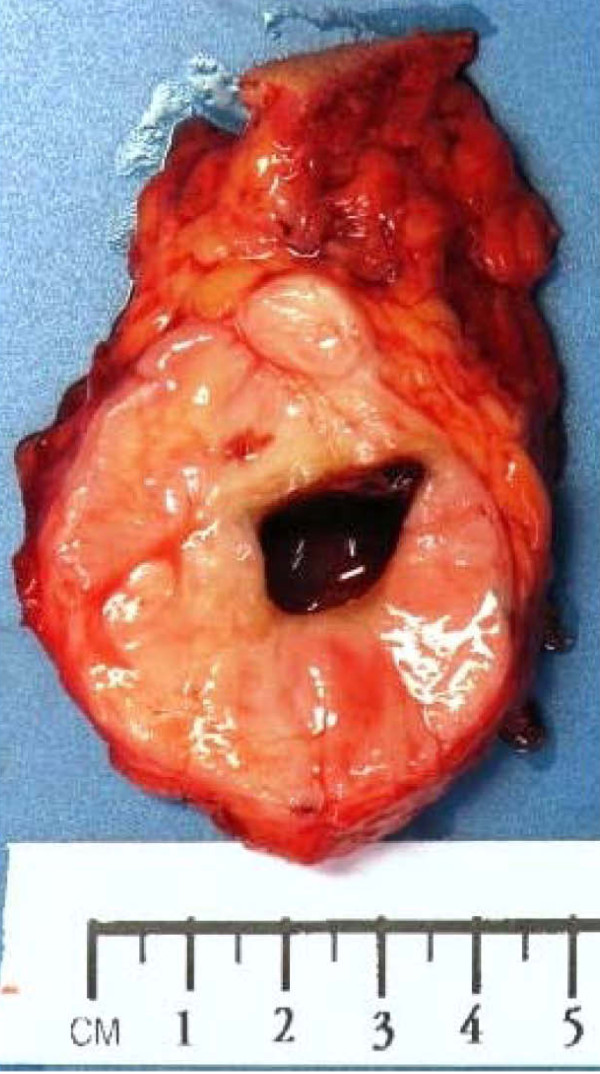
**Macroscopic appearances of a low grade fibromyxoid sarcoma tumour**.

**Figure 2:**
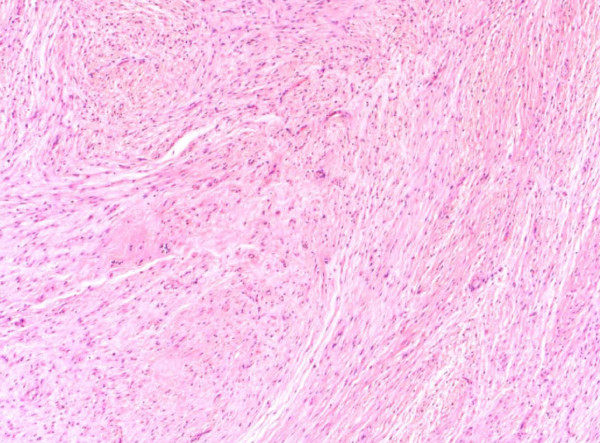
**Microscopic appearances of a low grade fibromyxoid sarcoma tumour (H&E stain, 40X magnification)**.

**Figure 3:**
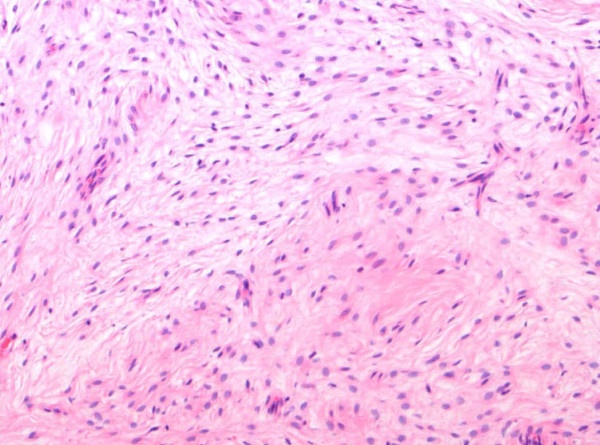
**Microscopic appearances of a low grade fibromyxoid sarcoma tumour (H&E stain, 100X magnification)**.

The histopathological diagnosis of the specimens, which were all undertaken by the senior Pathologist, was based on the light microscopic features. Histopathological examination revealed the classical features of LGFMS, which include a mixture of heavily collagenised zones and more cellular myxoid nodules. The tumour cells are classically spindle shaped and bland with occasionally scattered hyperchromatic cells and very scarce mitoses. Approximately 40% of LGFMS show focal poorly formed collagen rosettes which consist of a central core of hyalinized collagen surrounded by epithelioid fibroblasts [1] (Figure 1, Figure 2, Figure 3).

Specimens from a total of 21 patients underwent RT-PCR testing and 22 patients underwent FISH testing for the *FUS-CREB3L2 *translocation. Testing was performed solely on biopsy specimens in 8 patients (34.8%), solely on resection specimens in 10 patients (43.5%), and on both biopsy and resection specimens in 3 patients (8.7%).

RT-PCR testing was not performed on 2 specimens, and the RNA was inadequate for testing on 3 specimens. Tumours from 9 patients tested positively for the *FUS-CREB3L2 *transcript (50%) by RT-PCR, 2 tests were equivocal (11.1%), and 7 were negative (38.9%). Results are displayed in table 1.

**Table 1:** Comparison of FUS-CREB3L2 results

					*Location (%)*		
*FUS-CREB3L2 Test Result*	*Number (%)*	*Mean Age*	*M:F Ratio*	*Mean Maximum Diameter (mm)*	UL	LL	Trunk
RT-PCR Positive	50.0	34.0	2:1	99.7	11.1	55.6	33.3

RT-PCR Negative	38.9	47.1	1:2.5	64.1	42.9	28.6	28.6

FISH Positive	81.8	37.7	1:1.6	89.4	11.1	50	33.3

FISH Negative	18.2	51.0	1:1	66.3	75	25	0

M:F, Male: Female; UL, Upper Limb; LL, Lower Limb

The mean age of patients whose samples tested positive by RT-PCR was 34.0 years (range 14-52 years) compared to 47.1 years (range 24-70 years) for those testing negative (p = 0.10). Within the positive group, 5 tumours (55.6%) were located in the lower limbs, with 3 tumours (33.3%) in the trunk and 1 tumour (11.1%) in the upper limb. Within the group testing negative, 3 of the tumours (42.9%) were located in the upper limbs, compared to 2 (28.6%) in the trunk and 2 (28.6%) in the lower limbs. The mean maximum diameter for positive samples was 99.7 mm (range 30-150 mm), compared to 64.1 mm (range 20-130 mm) for those testing negative (p = 0.18).

FISH testing was not performed on 1 specimen. Eighteen patients tested *FUS-CREB3L2 *translocation positive (81.8%) and 4 were negative (18.2%). The mean age of patients whose samples tested positive by FISH was 37.7 years (range 10-67 years) compared to 51.0 years (range 31-70 years) for those testing negative (p = 0.21). Within the positive-testing group, 9 tumours (50%) were located in the lower limbs, with 6 tumours (33.3%) in the trunk, 2 tumours (11.1%) in the upper limbs and 1 tumour location unspecified (5.6%). Within the group testing negative, 3 of the tumours (75%) were located in the upper limbs and 1 (25%) in the lower limbs. The mean maximum diameter for positive samples was 89.4 mm (range 30-150 mm), compared to 66.3 mm (range 20-130 mm) for those testing negative (p = 0.44).

Three specimens tested negative by both RT-PCR and FISH. All of these specimens were further reviewed by the senior Pathologist to ensure that the histopathological diagnosis of LGFMS was correct.

## Discussion

Low-grade fibromyxoid sarcoma is a rare soft tissue neoplasm first described as a separate pathological entity by Evans in 1987 [1]. Its true incidence is unknown. Patients tend to be young to middle-aged adults. Evan's original series of 12 patients [5] reported ages ranging from 6 to 51 years, with all but three between 26 and 46 years of age. Further studies report mean ages of 38 years [13], 29 years [3], and 39 years (range 28 to 44 years) [4], and a median age of 34 years [12], and 45 years [2]. Our mean age of 40.6 years is comparable with these series. Our male: female ratio was 1:1.3 (10 male and 13 female patients). This is similar to the series reported by Guillou et al. [13] (22 male, 26 female). Folpe et al. [12] describe a male predominance (40 male, 33 female), as do the smaller series of Billings et al., Goodlad et al. and Zamecnik and Michal [2-4].

The largest series of LGFMS [12] reports 37 tumours located in the lower limb (including buttock and groin), 25 related to the trunk, head and neck, and 11 in the upper limb (including axilla). Guillou et al. [13] describe a ratio of 23:23:2, and combining other smaller published series [2-5] reveals a ratio of 25:17:7. Our series also shows the most common tumour location to be the lower limb, at a comparable ratio of 2:1.4:1.

The mean diameter of tumours reported by Billings et al. was 42 mm (range 16-160 mm) [3], with a median diameter reported as 45 mm (range 10-230 mm) by Folpe et al. [12], and 95 mm (range 35-150 mm) by Evans [5]. Our mean diameter was larger at 84.3 mm, but with a comparable median (87.5 mm) and range (20-150 mm).

We cannot account for the patients lost to follow-up. One patient was referred with a third recurrence of a para-spinal tumour, previously diagnosed as a neurofibroma. Our resection specimen showed the tumour to be a LGFMS. In retrospect, it is likely that this was a recurrence of a previously incorrectly-diagnosed LGFMS. When misdiagnosed, LGFMS is most commonly reported as a benign lesion, either a neurofibroma or a perineurioma. This may result in inadequate resection, thus increasing the chance of recurrence or metastasis. The tumour was excised with marginal margins, and the patient had post-operative radiotherapy. She has had no metastases to date, which represents 14 years following the original tumour resection.

In our series of patients in whom follow-up was achieved, no instances of local recurrence or metastasis occurred during the follow-up period, even though 12 patients (66.7%) had a marginal resection. This is substantially lower than other series, although we accept that our follow-up is medium-term. In their large series, Folpe et al. [12] report a local recurrence rate of 9%, metastasis rate of 6%, and 1% of cases dying of LGFMS at a mean of 38 months and median of 24 months follow-up. Guillou et al. [13] report a smaller series than Folpe, but with substantially longer follow-up. Their recurrence rate and metastasis rate were both 21% for those cases presenting with only local disease, with an overall metastasis rate of 27%. These are all significantly greater than the series reported by Folpe et al. Guillou et al reported over a much longer follow-up period, with the median times to local recurrence and metastasis being 276 months and 132 months respectively, and 83% of the cases of metastasis occurring beyond nine years follow-up. Billings et al. [3] followed 16 patients, experiencing 2 episodes of local recurrence (5 and 16 months), but no metastases. Goodlad et al. [2] reported a median follow-up of 6 years for 11 patients, experiencing 6 episodes of local recurrence and 1 patient with pulmonary metastases.

In our series the width of excision margin has had no impact on outcome to date, although Guillou et al. report that all their cases of local recurrence occurred following incomplete or marginal tumour excision [13].

Reaching the diagnosis of LGFMS can be difficult due to its bland-looking histological features. The differential diagnosis includes other benign or low-grade fibromyxoid lesions, including low-grade myxofibrosarcoma, myxoid neurofibroma, perineuroma, myxoid solitary fibrous tumour and desmoid fibromatosis [13,17]. Immunohistochemistry has produced some conflicting reports [17], and is therefore unreliable for confirming a diagnosis.

Cytogenetic and molecular genetic analyses have shown that many types of soft tissue sarcoma are characterised by specific chromosomal translocations resulting in 'chimeric fusion genes', which result in the production of chimeric transcription factors [16]. The *FUS *gene has been shown to be rearranged in a variety of neoplastic conditions, including myxoid liposarcoma, angiomatoid fibrous histiocytoma and acute myeloid leukaemia [16].

LGFMS was first characterised at a genetic level by Storlazzi et al. [16], who described 2 cases of a chromosomal translocation t(7;16)(q33;p11), which fuses the *FUS *gene to *BBF2H7 *(also known as *CREB3L2*). Panagopoulos et al. [15] subsequently suggested that the *FUS-CREB3L2 *translocation is specifically associated with LGFMS. They tested 59 tumours not previously identified as LGFMS for the *FUS-CREB3L2 *translocation. This test produced 12 positive specimens, all of which, upon histopatholgic re-examination, were diagnosed as LGFMS. In contrast however, Guillou et al. report that 7 out of 52 (13.5%) of their *FUS-CREB3L2 *fusion gene positive cases occurred in non-LGFMS neoplasms, of which 4 were diagnosed as sclerosing epithelioid fibrosarcoma [13]. This tumour may, in some instances, represent a morphologic variant of LGFMS, rather than a distinct entity in itself.

Guillou et al. report that 45 out of 59 LGFMS cases (76.3%) were positive for *FUS-CREB3L2 *[13]. Matsuyuma et al. report the identification of the *FUS-CREB3L2 *fusion gene in 88% of their LGFMS cases [11]. Their series identified the fusion gene solely in cases of LGFMS. Mertens et al. report this figure to be 96% in their series, and furthermore state that no other tumours were fusion-positive [14]. Our series produced a far lower number of positive tests by RT-PCR (50%), but a comparable number by FISH (81.8%). This would suggest that FISH testing is substantially more sensitive at detecting LGFMS than RT-PCR. Guillou et al. suggest that fusion-positive LGFMS have predominance in lower extremities (22/48 cases, 45.8%), which is in accordance with the 55.6% of RT-PCR positive cases and 50% FISH positive cases seen in our series [13].

We compared the clinical outcomes for those patients testing positive and negative for the *FUS-CREB3L2 *translocation using both RT-PCR and FISH. Despite not being statistically significant, negative-testing specimens occurred in older patients (47.1 years, as compared to 34.0 years for RT-PCR positive patients; 51.0 years, as compared to 37.7 years for FISH positive patients), and tended to be smaller (mean diameter 64.1 mm, as compared to 99.7 mm for RT-PCR positive patients; 66.3 mm, as compared to 89.4 mm for FISH positive patients). As the follow-up of our series revealed no patients with recurrence or metastasis, it is not possible to extrapolate as to whether a positive or negative test result for the *FUS-CREB3L2 *translocation has a bearing, or could be used as a predictive factor, for future patient morbidity or mortality. The tumour from the patient presenting with a third recurrence (previously incorrectly diagnosed as neurofibroma) tested positive by FISH. RT-PCR was not performed on this specimen.

The proteins encoded by *CREB3L1 *and *CREB3L2 *belong to the same family of transcription factors [14]. One study suggests that the *CREB3L2 *transcription factor is both functionally and structurally similar to the *CREB3L1 *transcription factor [20]. Therefore it is not surprising to find positive tests in cases of LGFMS for the *FUS-CREB3L1 *fusion-gene in the literature. The remaining case (4%) not testing positive for *FUS-CREB3L2 *in the series described by Mertens et al. tested positive for the *FUS-CREB3L1 *translocation, as did 3 cases (5.1%) in the series by Guillou et al. [13,14]. Our study did not test for the *FUS-CREB3L1 *translocation.

## Conclusion

In our series, the proportion of cases of LGFMS testing positive for the *FUS-CREB3L2 *translocation by RT-PCR is lower than previously described, but the proportion testing positive by FISH is comparable. Our results concur with previous data suggesting that the *FUS-CREB3L2 *fusion-gene is a specific marker for LGFMS. FISH testing is a more sensitive method of confirming a diagnosis of LGFMS.

Although not statistically significant, our series indicates that those tumours testing positive by RT-PCR or FISH tend to occur in younger patients and be larger in size. Patients followed-up in our series had no episodes of recurrence or metastasis post-operatively, although we identified one case where the tumour had previously been incorrectly diagnosed, and had subsequently recurred twice. The clinical outcome for both positive and negative-testing cases of LGFMS appears to be the same at medium-term follow-up. However, longer-term follow-up is required to elucidate whether the previously reported rates of late recurrence and metastasis are a true reflection of the biological nature of this tumour in our series, and may identify a difference in the long-term clinical outcome between translocation-positive and negative patients.

Our results suggest that with adequate surgery, local recurrence or metastasis is unlikely to occur in the short-term. Our medium-term results would theoretically suggest that it is not necessary to perform wide excision. However, due to the well-reported propensity for late recurrence or metastasis of LGFMS, we conclude that a wide excision is essential if surgically possible.

We conclude therefore that RT-PCR and FISH analysis for the *FUS-CREB3L2 *gene rearrangement are useful tools for confirming the diagnosis of LGFMS, but have no role in predicting the clinical outcome in the short and medium-term for such cases.

## Competing interests

The authors declare that they have no competing interests.

## Authors' contributions

BR and GT wrote, edited and revised the article. KD provided the pathological advice necessary for the paper. RP, JS, TB and SC provided the patients for the study and approved the final draft. All authors read and approved the final manuscript.
